# Nearly Full-Stokes Polarization Control Enabled by Geometric Polarization in Broadband Metasurfaces

**DOI:** 10.1021/acs.nanolett.5c02302

**Published:** 2025-06-24

**Authors:** Tzu-Yuan Lin, Shih-Hsiu Huang, Po-Chen Chen, Yu-Ching Lin, Chun-Ping Lin, Sung-Yu Chen, Pin Chieh Wu

**Affiliations:** † Department of Photonics, 34912 National Cheng Kung University, Tainan 70101, Taiwan; ‡ Advanced Photovoltaic and System Application Division, Green Energy and Environment Research Laboratories, 63129 Industrial Technology Research Institute, Tainan 711010, Taiwan; § Center for Quantum Frontiers of Research & Technology (QFort), 34912 National Cheng Kung University, Tainan 70101, Taiwan; ∥ Meta-nanoPhotonics Center, 34912 National Cheng Kung University, Tainan 70101, Taiwan

**Keywords:** Full-Stokes polarization control, Geometric polarization, Spectral tuning, Metasurfaces, Broadband polarization modulation, Anisotropic meta-atoms

## Abstract

The geometric phase has become a foundational element in modern metasurface design for optical phase and wavefront control. Here, we introduce the concept of geometric polarization, in which the orientation of the output polarization is modulated solely by in-plane rotation of anisotropic meta-atoms under circularly polarized illumination. Unlike conventional geometric phase approaches that manipulate converted circular polarization components, geometric polarization enables continuous tuning of the polarization azimuth on the Poincaré sphere while maintaining a fixed output ellipticity. Furthermore, with proper structural optimization, wavelength tuning modifies the amplitude and phase of the co-polarized component and the amplitude of the cross-polarized component, allowing the polarization states to span a broad latitudinal range. Together, geometric polarization and spectral tuning enable nearly full-Stokes polarization control using a single-layer metasurface. This work presents a conceptually distinct strategy for polarization engineering and expands the functional versatility of metasurfaces in optical modulation, imaging, and communication technologies.

## Graphical abstract

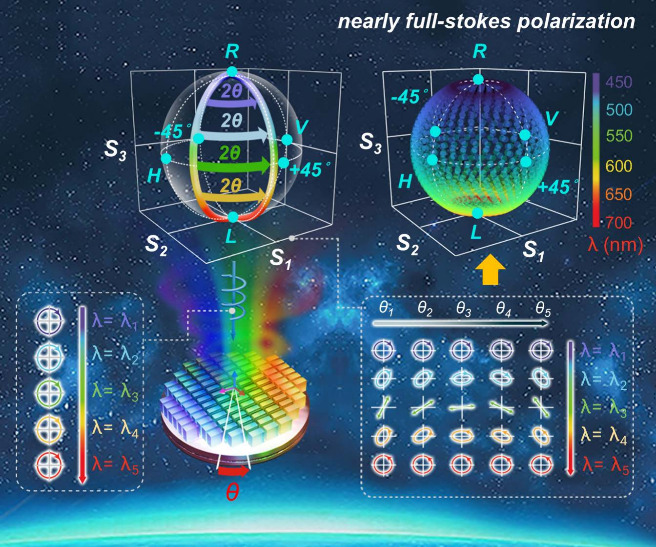

The geometric phase (also termed the Pancharatnam–Berry phase) is a fundamental concept in modern optics. It describes the phase shift that occurs when the polarization state of light evolves along a closed path in parameter space.^1^ Unlike the dynamic phase, it does not depend on the optical path length or refractive index. In recent years, metasurfaces have attracted significant attention in nanophotonics.^2^ By designing the shape, size, and orientation of each element, metasurfaces can control the amplitude, phase, and polarization of the light. Their compact and lightweight nature makes them a promising alternative to traditional bulky optical components.^3^ This has led to their widespread use in applications such as beam shaping,^4^ quantum photon generation,^5^ focusing and imaging,^6–8^ and optical information encryption.^9,10^

Among the many approaches used in metasurface design,^11–17^ the geometric phase has proven particularly effective and versatile. It specifically governs the phase of the orthogonal circularly polarized component of light. Under circularly polarized illumination, an anisotropic metasurface atom (hereafter referred to as the meta-atom) orientation rotated by an angle θ imparts a phase shift of ±2θ to the converted circular polarization component, depending on the input handedness.^18–20^ This unique characteristic makes the geometric phase a powerful mechanism for spin-dependent phase control.^21^ When the coupling between elements is weak, the scattering amplitude remains nearly constant during rotation; therefore, the far-field intensity stays stable as the phase varies continuously. These features make the geometric phase a highly efficient and broadband-compatible mechanism for use in metasurface-based optical devices, such as metalenses,^22,23^ beam deflectors,^18^ and holographic displays.^24^ In many cases, the design freedom provided by the geometric phase has also been combined with other mechanisms, such as the propagation phase^25–29^ and optical chirality,^30–33^ to achieve enhanced functionality, symmetry breaking, or multiplexing.

While the concept of the geometric phase has been extensively studied and has greatly contributed to the advancement of metasurface optics,^34^ most existing implementations are still constrained by a prevailing design paradigm. Typically, the geometry and dimensions of individual meta-atoms are optimized to maximize cross-polarization conversion efficiency at a designated center wavelength^35^ or within a limited spectral band, primarily for wavefront shaping applications.^36,37^ Specific optical functionalities, particularly polarization conversion, are typically implemented by spatially distributing optimized meta-atoms in interleaved or segmented configurations across the metasurface.^38–42^ Although this strategy has proven to be effective in achieving discrete polarization states, it introduces fundamental limitations. As the number of target polarization channels increases, the metasurface area must be divided among more domains, which often leads to a reduction in the optical efficiency per channel. Moreover, spatial multiplexing strategies tend to introduce discontinuities in both phase and amplitude distributions, complicating the optical design and fabrication processes and limiting the scalability of such approaches. To overcome these limitations, several prior works have explored multiwavelength metasurfaces in which different incident wavelengths are used to activate distinct polarization states from a single device structure.^43,44^ While these approaches represent an important step toward spectral multiplexing, the number of achievable polarization states remains limited, and the resulting modulation is typically discrete rather than continuous. A more generalized framework that decouples polarization manipulation from such constraints and enables dynamic and continuous modulation of polarization states, ultimately supporting full-Stokes polarization control within a single metasurface platform, is still missing.

In this study, we introduce a refined interpretation of the geometric phase by examining the full evolution of the output polarization state on the Poincaré sphere. Instead of focusing only on the converted circularly polarized component, we consider both co- and cross-polarized contributions and analyze how their coherent superposition determines the resulting polarization. When the metasurface is illuminated with circularly polarized light, rotating the anisotropic meta-atom leads to a linear change in the azimuthal angle of the output polarization while maintaining a constant output ellipticity. We refer to this effect as geometric polarization, as it arises directly from the physical rotation of the nanostructure. By further tuning the incident wavelength, we control the amplitude and phase of the co-polarized component and the amplitude of the cross-polarized one. This spectral dependence allows the polarization state to span a wide range along the latitude of the Poincaré sphere. Together, geometric polarization and wavelength tuning enable continuous access to a broad range of polarization states. These findings demonstrate that a single, noninterleaved metasurface composed of identical meta-atoms can support nearly full-Stokes polarization modulation, providing a compact and reconfigurable platform for advanced optical control.

To provide a rigorous theoretical foundation for the proposed concept of geometric polarization, we present two complementary formulations that describe how the output polarization evolves under structural rotation and spectral tuning. We first analyzed the system by using the Jones matrix formalism. When a circularly polarized wave is incident on an anisotropic meta-atom, the reflected field can be expressed as a superposition of co- and cross-polarized circular components, with complex reflection amplitudes *r*_*co*_ and *r*_*cross*_, respectively. The co-polarized term preserves the input helicity, while the cross-polarized term reverses it and accumulates a geometric phase 2θ, where θ is the in-plane rotation angle of the meta-atom. The total reflected field is given by

1 $$\left[\begin{array}{l} E_{x}^{out}\\ E_{y}^{out} \end{array}\right]=\frac{r_{co}}{\sqrt{2}}\left[\begin{array}{l} 1\\ i \end{array}\right]+\frac{r_{cross}}{\sqrt{2}}e^{i2\theta }\left[\begin{array}{l} 1\\ -i \end{array}\right]$$

where *E*_*i*_^*out*^ represents the *i*-component of the total output electric field (*i* = *x*, *y*). From this expression, we derive the corresponding Stokes parameters using their standard definitions:

2 $$S_{0}={\left|r_{co}\right|}^{2}+{\left|r_{cross}\right|}^{2}$$

3 S1=|Exout|2−|Eyout|2=2Re[rco·rcrosse−i2θ]

S2=2Re{Exout×(Eyout)*}=2Im[rco·(rcross)*e−i2θ] 4

5 S3=−2Im{Exout×(Eyout)*}=(|rco|2−|rcross|2)

Here, (*E*_*y*_^*out*^)* and (*r*_*cross*_)* indicate the complex conjugate of *E*_*y*_^*out*^ and *r*_*cross*_, respectively. These equations reveal that *S*_1_ and *S*_2_ are modulated by both the relative phase between the two components and the meta-atom rotation angle θ, while *S*_3_ depends only on the intensity difference and remains invariant with respect to the orientation. In addition to angular modulation, the reflection coefficients *r*_*co*_ and *r*_*cross*_ vary with the incident wavelength due to the dispersive response of the meta-atom. By designing the meta-atom geometry appropriately, the amplitudes and phases of these components can be spectrally engineered so that sweeping the wavelength can potentially shift the polarization state along the meridional direction of the Poincaré sphere. This spectral tuning, combined with geometric rotation, potentially enables control over both azimuthal and polar coordinates of the polarization state, providing access to nearly the full surface of the Poincaré sphere and thus realizing full-Stokes polarization control using a single metasurface (see Part 1 of the Supporting Information for more details).

To further explain the geometric nature of the rotation-induced polarization modulation, we consider the behavior of the output electric field in the rotated coordinate frame. When the meta-atom is rotated by an angle θ, the electric field transforms as

6 $$\left(\begin{array}{l} E_{x}^{\prime }\\ E_{y}^{\prime } \end{array}\right)=\left(\begin{array}{ll} \cos\theta & -\sin\theta \\ \sin\theta & \cos\theta \end{array}\right)\left(\begin{array}{l} E_{x}\\ E_{y} \end{array}\right)$$

By substituting these rotated field components into the Stokes parameter definitions, we obtain the transformed Stokes parameters *S*_1_^′^, *S*_2_^′^, and *S*_3_^′^. The result yields the following expressions:

7 $$S_{1}^{\prime }=S_{1}\;\cos2\theta -S_{2}\;\sin2\theta$$

$$S_{2}^{\prime }=S_{1}\;\sin2\theta +S_{2}\;\cos2\theta$$ 8

9 $$S_{3}^{\prime }=S_{3}$$

This transformation shows that the linear polarization components *S*_1_ and *S*_2_ rotate within the Stokes space by an angle of 2θ while leaving *S*_3_ unchanged. The azimuthal angle φ of the output polarization transforms as

10 $$\varphi^{\prime }=\tan^{-1}\left(\frac{S_{2}^{\prime }}{S_{1}^{\prime }}\right)=\varphi +2\theta$$

where φ and φ′ are the azimuthal angles before and after rotating the meta-atom, respectively. This result confirms a direct and linear relationship between the meta-atom rotation angle and the polarization orientation, and it holds independent of the ellipticity of the output polarization state. More details of the derivation process can be found in Part 2 of the Supporting Information.

To highlight the distinction between geometric phase and geometric polarization, Figure 1 presents their respective polarization evolution paths on the Poincaré sphere under circularly polarized illumination. In the geometric phase framework (left panel in Figure 1), when an anisotropic meta-atom is rotated by an angle θ, the polarization conversion occurs exclusively between orthogonal circularly polarized states. This results in a phase shift of ±2θ in the converted polarization component, while the polarization state itself traces a closed path. Notably, this process alters the phase but retains the circular polarization handedness of the input and output as mirror opposites.

**Figure 1:**
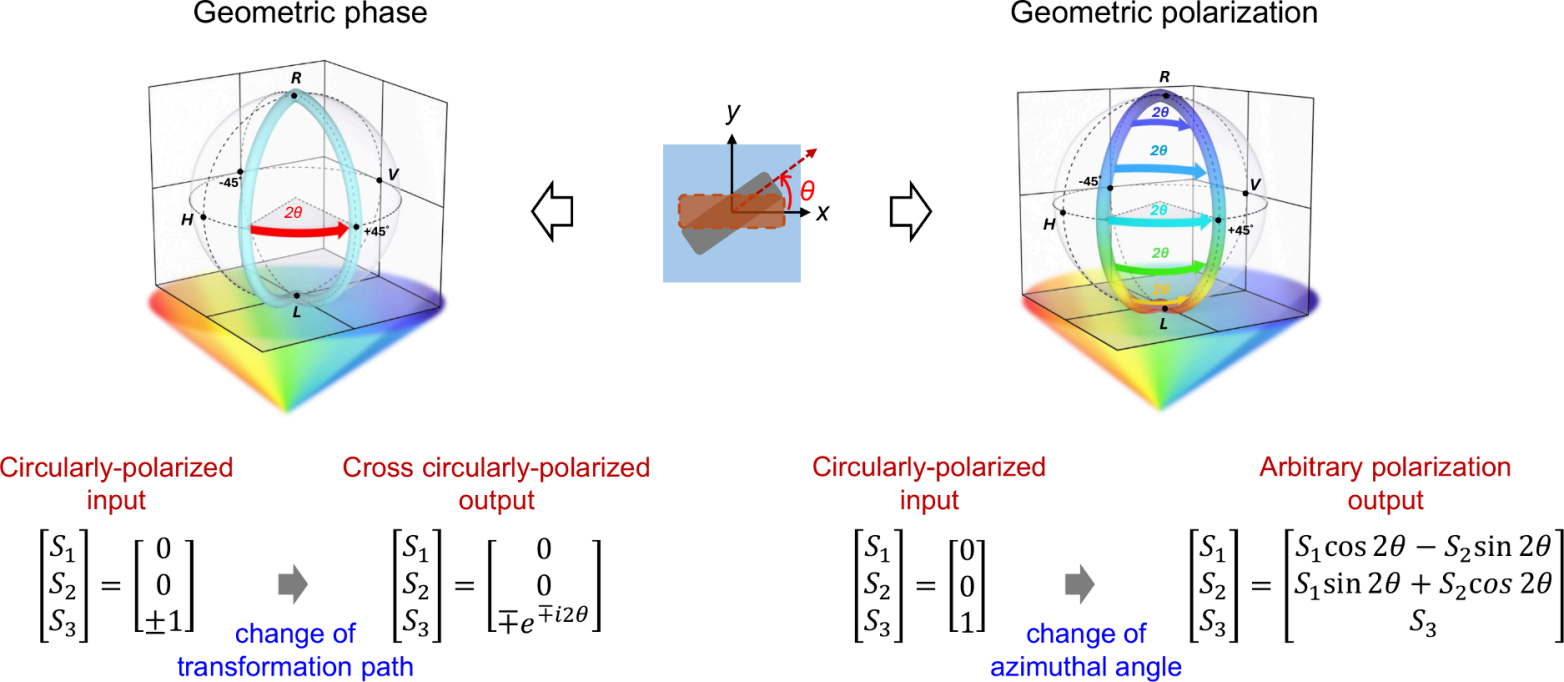
Schematic illustration of geometric phase and geometric polarization. The left panel shows the geometric phase, where the circularly polarized input leads to a cross circularly polarized output after interacting with an anisotropic metasurface. A phase shift of ±2θ occurs when the metasurface is rotated by an angle θ, with the sign depending on the polarization states of the input and output beams. A +2θ shift corresponds to a RCP to LCP transition, while a −2θ shift corresponds to the reverse. The right panel illustrates the concept of geometric polarization, in which the output polarization moves along a constant-latitude circle on the Poincaré sphere as the metasurface orientation varies under circularly polarized illumination. Different colors represent different incident wavelengths, demonstrating that the output polarization state is wavelength-dependent. The azimuthal angle of the polarization vector changes due to structure rotation, highlighting the unique behavior of geometric polarization across the entire Poincaré sphere. The rainbow-colored cones illustrate that both the geometric phase and the proposed geometric polarization remain dispersionless, maintaining their effects across different wavelengths of incidence.

In contrast, our proposed geometric polarization concept (right panel in Figure 1) offers a broader and more intuitive interpretation of the polarization transformation. Instead of focusing solely on the converted component, we examine the evolution of the output polarization as a continuous function of the rotation of meta-atoms. By fixing the geometry and resonance condition of the nanostructure, the rotation of meta-atoms induces a deterministic change in the azimuthal angle of the output polarization (as theoretically discussed above). This effect manifests as a continuous trajectory on the Poincaré sphere, not constrained to any output polarization state.

To experimentally validate the geometric polarization behavior predicted by our analytical model, we fabricated a metasurface composed of aluminum (Al) nanoantennas positioned on a dielectric spacer above a reflective substrate (see Methods in the Supporting Information for fabrication details), as illustrated in the right panel of Figure 2a. By optimizing the geometric dimensions of the meta-atoms, the phase and amplitude differences between the co- and cross-polarized circularly reflected components can be controlled. The interference between these components produces reflected light with tunable polarization states. Therefore, the anisotropic meta-atoms were designed to operate under circularly polarized illumination and generate wavelength-dependent polarization states upon reflection. Both numerical simulations and full-Stokes parameter measurements (see Figure S1 for details of optical setup) were performed across the visible spectral range (450–700 nm). As shown in Figure 2a, the simulated (solid lines) and measured (dotted dots) Stokes parameters exhibit consistent spectral trends, confirming that the polarization state of the reflected light evolves continuously with wavelength. To visualize this polarization evolution in full Stokes space, Figure 2b shows the trajectory of the polarization state on the Poincaré sphere as a function of the wavelength. Both the simulated (left image in Figure 2b) and measured (right image in Figure 2b) results clearly demonstrate a continuous progression of the output polarization state with increasing wavelength. Notably, the polarization vector traces a smooth trajectory along the longitudinal direction of the sphere, spanning from near right-handed circular polarization (RCP) to near left-handed circular polarization (LCP) as the wavelength varies from 450 to 700 nm. This broad polarization modulation range is beneficial for verifying the versatility of the proposed geometric polarization mechanism. Without appropriate optimization of the meta-atom geometry, tuning the incident wavelength may not result in a substantial variation in the output polarization states (refer to Figure S2).

**Figure 2:**
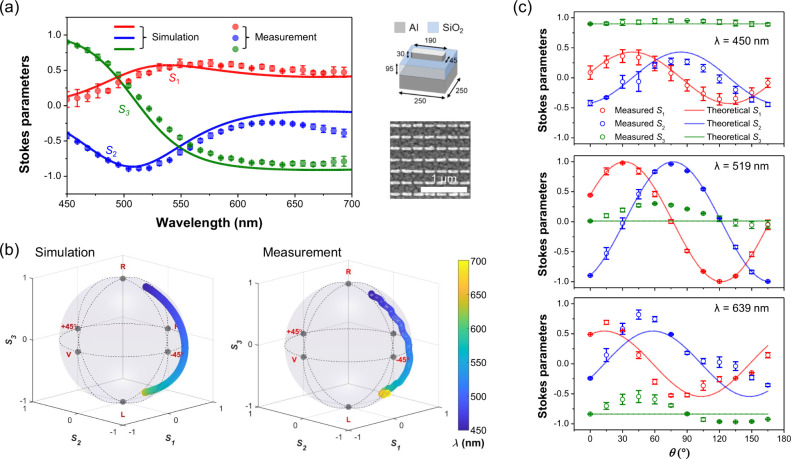
(a) Simulated (solid curves) and measured (dots) Stokes parameters *S*_1_, *S*_2_, and *S*_3_ as a function of wavelength. The right panels display a schematic of the metasurface structure (unit: nm) and a corresponding SEM image of the fabricated sample. (b) 3D plots of the simulated and measured Stokes parameters on the Poincaré sphere, illustrating the polarization evolution as the incident wavelength varies. The color scale represents the wavelength range from 450 to 700 nm. (c) Stokes parameter profiles at selected wavelengths (λ = 450, 519, and 639 nm), comparing the measured values (circles) and theoretical predictions (curves) at different structural orientation angles. Minor oscillations observed in the measured *S*_3_ parameter at certain wavelengths may result from residual interference in the optical setup and minor imperfections in the fabricated samples. To minimize sample-dependent variations, measurements from three independent devices were averaged, and the standard deviation is represented as error bars. All simulations were conducted by rotating individual meta-atoms about their central axes.

Further validation is provided in Figure 2c, which presents the measured and theoretical output Stokes parameters as a function of the structural rotation angle θ at three representative wavelengths. Additional data are provided in Figure S3. For simplicity, different structural orientations are implemented by rotating the entire metasurface sample in the experiment. This approach yields an equivalent effect to rotating individual meta-atoms, provided that near-field coupling between adjacent elements is negligible, which holds true under the design conditions of this work (refer to Figure S4 for more details). As can be seen in Figure 2c, the measured *S*_1_ and *S*_2_ values exhibit a clear sinusoidal dependence on 2θ at all wavelengths, in excellent agreement with the theoretical predictions based on eqs 7 and 8. In contrast, *S*_3_ remains nearly constant across all θ values, as expected from theoretical calculations. The observed sinusoidal variation of *S*_1_ and *S*_2_, combined with the θ-invariant and nearly wavelength-independent behavior of *S*_3_, confirms that the output polarization undergoes rotation at a fixed ellipticity when the incident wavelength is held constant and the metasurface orientation is varied.

To further elucidate the polarization transformation characteristics introduced by geometric polarization, we analyze the behavior of the output polarization state across both the wavelength and rotation angle domains. Figure 3a schematically illustrates the azimuthal angle φ of the polarization state on the Poincaré sphere, which serves as a key metric for characterizing the output polarization orientation along the latitude directions. Figure 3b presents a comprehensive φ-mapping as functions of both the structural rotation angle θ and the wavelength, comparing numerical simulations with experimental measurements. In both cases, the output azimuthal angle exhibits a linear dependence on the rotation angle θ, with the color gradient indicating a smooth and continuous increase in φ with increasing θ. This behavior is consistently observed across the full spectral range from 450 to 700 nm, confirming the broadband, wavelength-independent nature of geometric polarization. The simulated results obtained by rotating the entire metasurface (refer to Figure S5) closely match those presented in Figure 3b, further confirming that in this work, rotating the entire metasurface yields an optical response equivalent to that of rotating individual meta-atoms.

**Figure 3:**
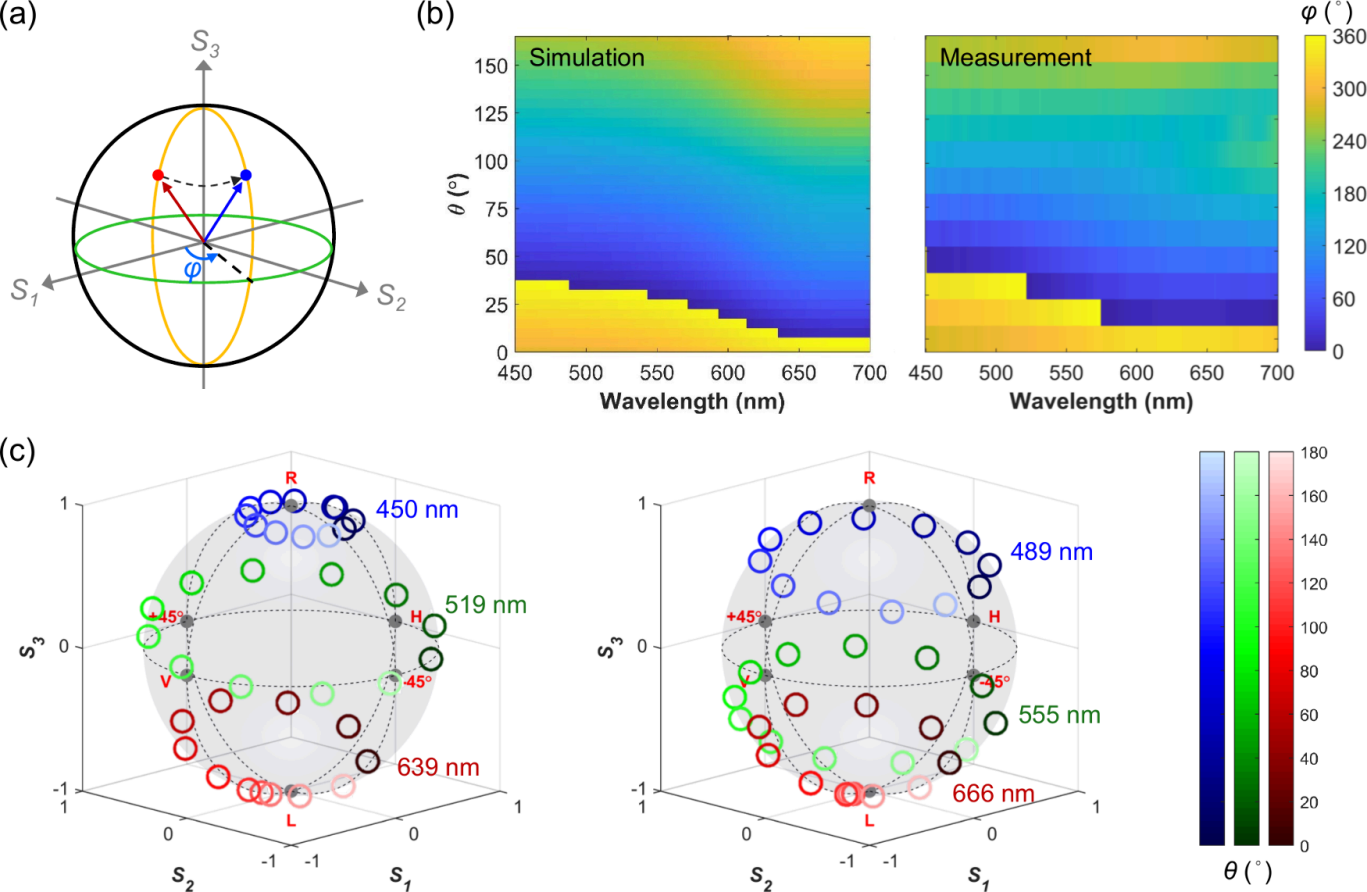
(a) Schematic illustration of polarization state evolution on an azimuthal plane. Dark red and blue arrows represent the polarization vectors of reflected beams corresponding to different structural orientation angles. (b) Simulated and measured azimuthal angle as a function of both wavelength and structural orientation angle θ. (c) Stokes parameter distribution of the reflected light under varying wavelength conditions, highlighting the wavelength-independent nature of polarization orientation control enabled by geometric polarization. The numerical simulations were performed by rotating individual meta-atoms around their central axes. All experimental results were obtained by averaging measurements from three samples.

To visualize the trajectory of polarization states in 3D Stokes space under multiple conditions, Figure 3c plots the measured polarization states on the Poincaré sphere at various wavelengths and rotation angles. The data obtained at different wavelengths form distinct circular arcs around the *S*_3_ axis as the rotation angle θ increases from 0° to 180° (see Figure S6 for additional experimental results). These arcs lie on latitude planes determined by the polarization ellipticity, which remains nearly constant at all wavelengths, as evidenced by the preserved *S*_3_ values. Meanwhile, the azimuthal angle φ of the polarization vector varies continuously with θ, forming a closed loop on the latitude plane as θ sweeps from 0° to 180°. This behavior results in the appearance of concentric rings on the Poincaré sphere, consistent with the 2θ modulation law.

To evaluate the practical viability of geometric polarization for optical device applications, we investigated the reflection efficiency of our metasurface under various structural rotational angles across the spectral range of interest. Figure 4a presents the simulated and experimentally measured reflection spectra for rotation angles θ ranging from 0° to 150°. At all rotation states, the metasurface exhibits moderate to high reflectance (typically exceeding 50%) across the full wavelength range from 450 to 700 nm. These results confirm that the polarization transformation enabled by geometric polarization is achieved with sufficient optical efficiency. Importantly, the reflection remains relatively stable as the rotation angle varies, which is a key attribute for practical applications where polarization modulation is realized through metasurface rotation. This behavior is expected, as the geometric polarization mechanism operates under circularly polarized illumination and all meta-atoms share identical geometric dimensions. Although minor discrepancies are observed between the simulation and experiment, the overall spectral trends and reflectance levels show good agreement. These results confirm that high reflection is maintained, indicating that polarization control through geometric polarization and spectral tuning can be achieved without sacrificing optical efficiency.

**Figure 4:**
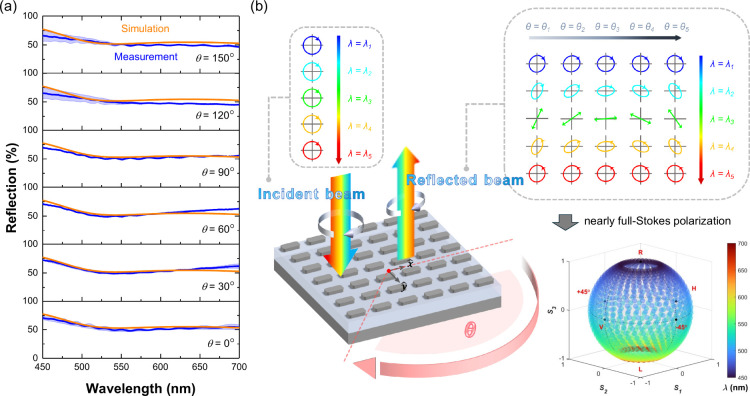
(a) Simulated and measured reflection spectra for various metasurface orientation angles (θ = 0° to 150°). The experimental results represent the average values obtained from three fabricated metasurface samples. The light blue shaded regions indicate the corresponding standard deviation. (b) Illustration of the polarization modulation capability enabled by geometric polarization in metasurfaces. With the incident polarization fixed as RCP across all wavelengths, the polarization state of the reflected light can be tuned by adjusting both the metasurface orientation and the incident wavelength. In this demonstration, the helicity of the input light is arbitrarily chosen. Equivalent results can be obtained using the opposite helicity. The lower-right panel presents a 3D plot of the simulated Stokes parameters (with a wavelength step of 5 nm), demonstrating broadband and continuous polarization modulation. Again, the numerical simulations were performed by rotating individual meta-atoms around their central axes.

The conceptual illustration in Figure 4b further clarifies the working principle and application potential of the proposed concept. Customized polarization states of the reflected light can be generated by rotating the anisotropic meta-atoms or the entire metasurface relative to the incident beam and tuning the incident wavelength. The top-right inset shows a 2D mapping of output polarization states on the Poincaré sphere as functions of metasurface orientation angle θ (horizontal axis) and incident wavelength λ (vertical axis). When the incident beam is fixed as RCP, the polarization state of the reflected light can be tailored by appropriately selecting the incident wavelength. Upon rotation of the metasurface, the orientation of the reflected polarization state can be continuously modulated, regardless of its initial ellipticity or handedness. The smooth and continuous evolution of polarization states across the 2D array demonstrates the capability of the design to achieve spatially varying and broadband polarization distributions. It is worth noting that the polarization orientation in this demonstration changes in a clockwise direction; however, the rotation sense can be reversed depending on the combination of the incident polarization helicity and the direction of the structural orientation change. Because the ellipticity is governed by the incident wavelength and the azimuthal angle is controlled by metasurface rotation, the system possesses two independent and complementary tuning parameters. This dual modulation capability makes it possible to achieve full-Stokes polarization control using a single metasurface. To further support this conclusion, we performed numerical simulations from 450 to 700 nm and plotted all output polarization states on the Poincaré sphere, as shown in the bottom-right panel of Figure 4b. The distribution of data points confirms that the combined control of wavelength and orientation allows the reflected polarization to span nearly the entire sphere, thereby validating the feasibility of achieving broadband and continuous full-Stokes polarization control with a simple and uniform metasurface design.

Since the incident beam is slightly focused during the optical measurements (see Figure S1), we finally evaluated the angular robustness of the proposed mechanism. To this end, we performed full-wave simulations of the Stokes parameters and the output azimuthal angle under various angles of incidence. Given that the numerical aperture of the objective lens used in our optical setup is 0.28, the maximum incident angle under tight focusing conditions is ∼16.26°. In our simulations, we characterized the optical performance of the proposed metasurface up to an incident angle of 20°, which slightly exceeds the experimental condition. As shown in Figure S7, the simulated Stokes parameters remain consistent across the visible spectrum for incident angles ranging from 0° to 15°. When the incident angle increases to 20°, only minor deviations are observed in the spectral trends and amplitudes of the Stokes parameters, indicating that the polarization response is largely maintained within this angular range. Additional evidence is provided in Figure S8, which presents simulated azimuthal angles φ as functions of wavelength and structural rotation angle θ for several incident angles. The resulting color maps demonstrate smooth and continuous modulation of the azimuthal angle with nearly identical patterns across all tested angles of incidence. This strongly supports the conclusion that the output polarization orientation follows the same rotation-induced trajectory on the Poincaré sphere regardless of moderate variations in the angle of incidence.

In summary, this work extends the conventional concept of the geometric phase by introducing and experimentally validating the idea of geometric polarization: a rotation-induced modulation of the polarization state originating from the angular orientation of anisotropic meta-atoms. Distinct from traditional resonance-based methods, this mechanism enables continuous and broadband control of the polarization azimuthal angle. Through analytical derivation based on Stokes parameter transformations, we demonstrated that rotating the metasurface results in a predictable azimuthal shift of the polarization state that is linearly proportional to the rotation angle. Unlike conventional full-Stokes or vectorial metasurfaces that typically require spatial multiplexing of meta-atoms with varying geometries,^45–47^ the approach demonstrated in this work enables nearly full-Stokes polarization control by simply rotating identical anisotropic structures under broadband illumination. The proposed geometric polarization mechanism preserves the ellipticity of the output polarization state while allowing independent and continuous tuning of its azimuthal angle. This decoupling between azimuthal orientation and ellipticity provides greater flexibility in tailoring the polarization states across the Poincaré sphere. We emphasize that the concept of geometric polarization extends beyond conventional geometric phase modulation by describing the complete evolution of the polarization state rather than focusing solely on the phase shift of the converted polarization component. In previous metasurface-based waveplate studies, advanced polarization control functionalities are often achieved by tuning anisotropic resonances or combining geometric and propagation phases, with the propagation phase typically introduced by altering the physical dimensions of the meta-atoms.^28,42^ Moreover, these approaches generally target a specific output polarization state, requiring external polarizers to isolate the desired component from the scattered light. In contrast, our method employs a fixed structural geometry and uses wavelength tuning to modulate the propagation phase in the copolarized channel. Since the output polarization state in our design arises from the coherent superposition of co- and cross-polarized components, no additional optical filtering elements are needed.

Finally, distinct from previous methods whose performance tends to degrade when scaled to multiple polarization channels, the proposed approach maintains a high operational efficiency regardless of the number of targeted polarization states. This robustness originates from the fact that polarization control in our design is achieved purely through geometric rotation and wavelength of incidence, without the need for segmented or interleaved meta-atom arrangements that often introduce spatial discontinuities and reduce the optical efficiency. This energy-efficient feature is essential for practical implementation, particularly in systems that require dynamically varying polarization profiles. Given that the proposed geometric polarization mechanism is effective across a broad spectral range, the design concept could potentially be extended to shorter wavelengths, such as the ultraviolet regime,^48,49^ or adapted to transmission-mode configurations using dielectric materials as building blocks. With appropriate material selection and optimized nanofabrication strategies, this approach holds promise for future applications in UV polarization optics and biospectroscopic systems. As supported by the generalized derivations in Parts 1 and 2 in the Supporting Information, the underlying mechanism is geometry-driven and independent of material refractive index, suggesting that similar polarization control can be realized using dielectric metasurfaces in transmission mode (also refer to Figure S13). This flexibility opens a promising route for implementing polarization-encoded imaging, broadband polarization beam shaping, and multifunctional metasurface platforms.

## Supplementary Material



## Data Availability

Data underlying the results presented in this paper are not publicly available at this time but may be obtained from the authors upon reasonable request.

## Notes

Schematic of optical setup; structural dimensions dependence on polarization modulation angle; experimental and theoretical Stokes parameter profiles at selected wavelengths; simulated electric field intensity distributions under different meta-atom orientations; simulated polarization modulation by rotating the entire metasurface; Stokes parameter distribution of the reflected light on the Poincaré sphere under varying wavelength conditions; simulated Stokes parameters as functions of wavelength and incident angle; simulated and measured azimuthal angles as functions of both wavelength and structural rotation angle under various angles of incidence; experimentally measured refractive index of Al and SiO_2_; experimentally measured refractive index of Al and optical index obtained from references; numerically calculated Stokes parameters of the proposed metasurface using different optical indices; 3D plots of the simulated Stokes parameters on the Poincaré sphere using different optical indices; demonstration of nearly full-Stokes polarization modulation using an all-dielectric metasurface in transmission; derivation of Stokes parameter transformation under structural rotation with Jones matrix; transformation of Stokes parameters and azimuthal angle under structural rotation with the rotated coordinate frame; and Methods section (PDF)
