# Supplementary material for: Nearly Full-Stokes Polarization Control Enabled by Geometric Polarization in Broadband Metasurfaces
Source: Nano Lett. 2025 Jun 24;25(26):10579–87. doi: 10.1021/acs.nanolett.5c02302 (PMC12232375; doi:10.1021/acs.nanolett.5c02302)
Supplement: Supplementary file 1 [file nl5c02302_si_001.pdf]

Supporting Information

Nearly Full-Stokes Polarization Control Enabled by Geometric Polarization in Broadband Metasurfaces

Tzu-Yuan Lin[1], Shih-Hsiu Huang[1], Po-Chen Chen[1], Yu-Ching Lin[1], Chun-Ping Lin[1], Sung-Yu Chen[2], and Pin Chieh Wu[1,3,4*]

[1]Department of Photonics, National Cheng Kung University, Tainan 70101, Taiwan

[2]Advanced Photovoltaic and System Application Division, Green Energy and Environment Research Laboratories, Industrial Technology Research Institute, Tainan 711010, Taiwan

[3]Center for Quantum Frontiers of Research & Technology (QFort), National Cheng Kung University, Tainan 70101, Taiwan

[4]Meta-nanoPhotonics Center, National Cheng Kung University, Tainan 70101, Taiwan

[*]E-mail address: pcwu@gs.ncku.edu.tw

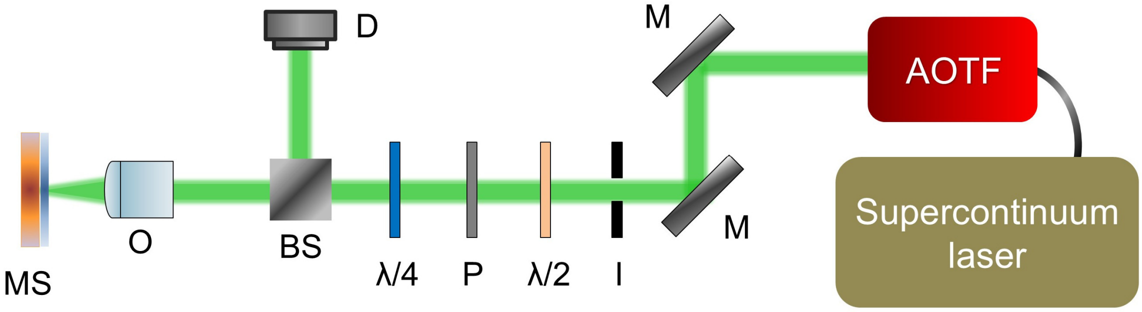

Figure S1. A supercontinuum laser (NKT Photonics FIU-15), in combination with an acousto-optic tunable filter (AOTF, SuperK SELECT), was used to select wavelengths within the visible range. For Stokes parameter measurements, a visible polarimeter (Thorlabs PAX1000VIS) served as the detector. In measurements of the metasurface reflection intensity, a power meter was used instead. To define the incident polarization state, a half-wave plate (Thorlabs AHWP05M-600), a linear polarizer (Thorlabs LPVISE100-A), and a quarter-wave plate (Thorlabs AQWP05M-600) were employed. The optical components used in the setup are denoted as follows: M: mirror; I: iris; $\lambda/2$: half-wave plate; P: linear polarizer; $\lambda/4$: quarter-wave plate; O: objective (Mitutoyo 10× magnification with 0.28 numerical aperture); D: detector.

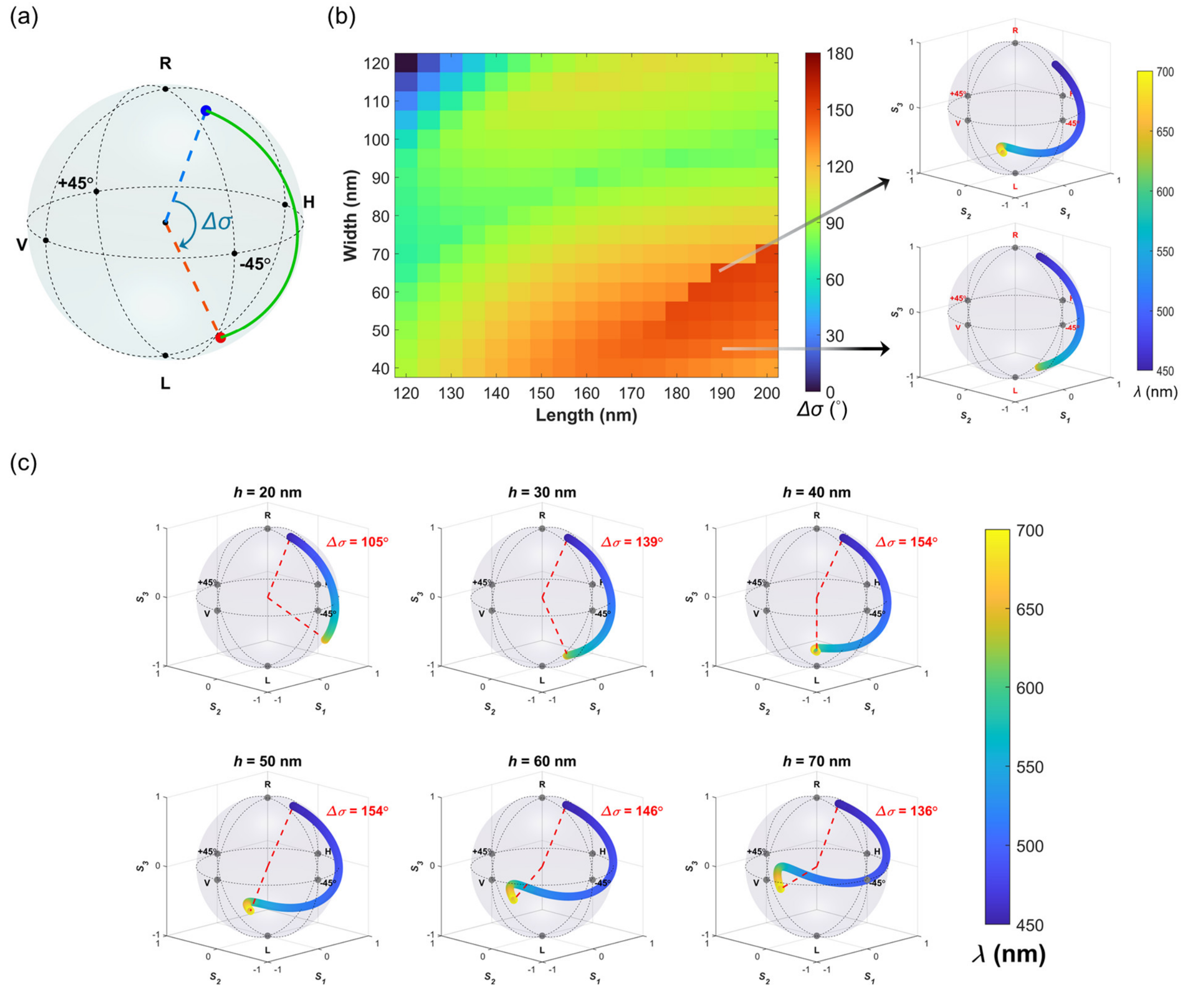

Figure S2. (a) Schematic illustration of the definition of the polarization modulation angle $\Delta\sigma$, which is determined by the angular separation between the northernmost and southernmost polarization states mapped on the Poincaré sphere under wavelength tuning. A larger $\Delta\sigma$ indicates a broader achievable range of polarization modulation. (b) Left panel: Calculated polarization modulation angle $\Delta\sigma$ as a function of meta-atom length and width under fixed RCP illumination. The maximum $\Delta\sigma$ is observed to be near 190 nm length and 65 nm width. Right panel: Simulated polarization trajectories on the Poincaré sphere for two selected geometries, highlighting how polarization states evolve with wavelength. The 190 nm × 65 nm structure exhibits a larger $\Delta\sigma$ but deviates from the ideal longitudinal path at longer wavelengths, while the 190 nm × 45 nm structure provides a more continuous and symmetric polarization evolution. (c) Simulated polarization trajectories on the Poincaré sphere for various thicknesses of the Al meta-atom. Among all cases, the polarization states span the broadest area of the sphere when the thickness h = 30 nm, which corresponds to the design parameter used in this work.

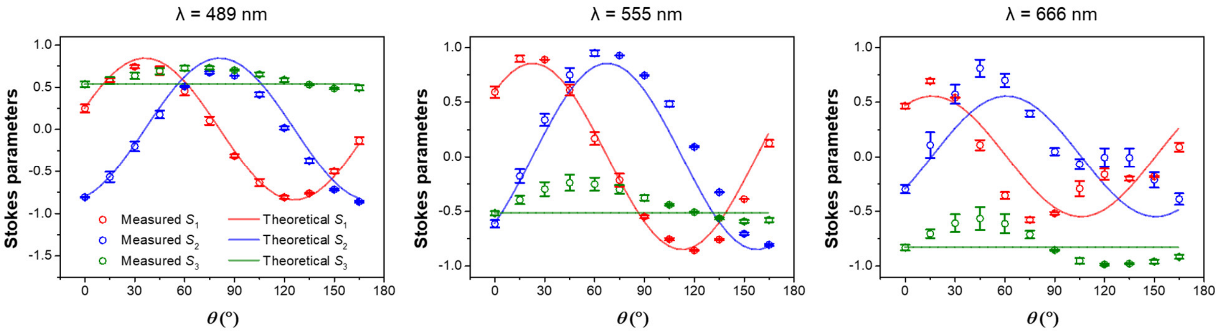

Figure S3. Stokes parameter profiles at selected wavelengths (λ = 489, 555 and 666 nm), comparing the measured values (circles) and theoretical predictions (curves) at different structural orientation angles. The experimental results were obtained by averaging measurements from three samples, with the errors represented by the standard deviation.

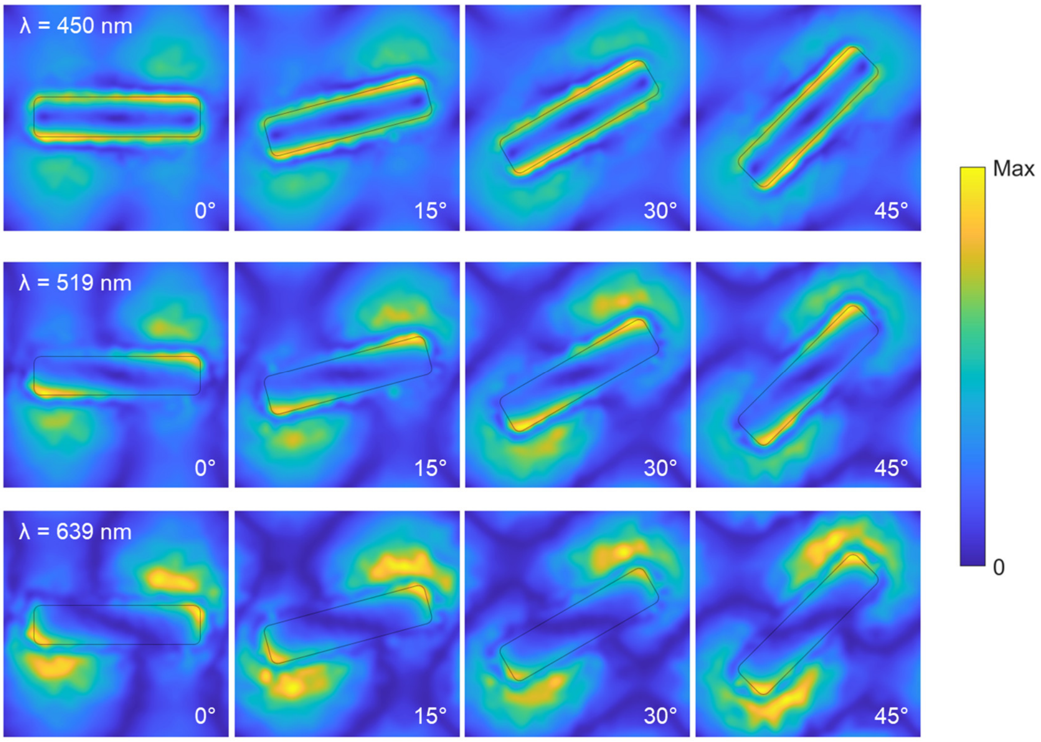

Figure S4. Simulated electric field intensity distributions under different meta-atom orientations show that the field energy remains strongly localized around individual nanostructures. This confirms that near-field coupling is negligible across all rotation angles.

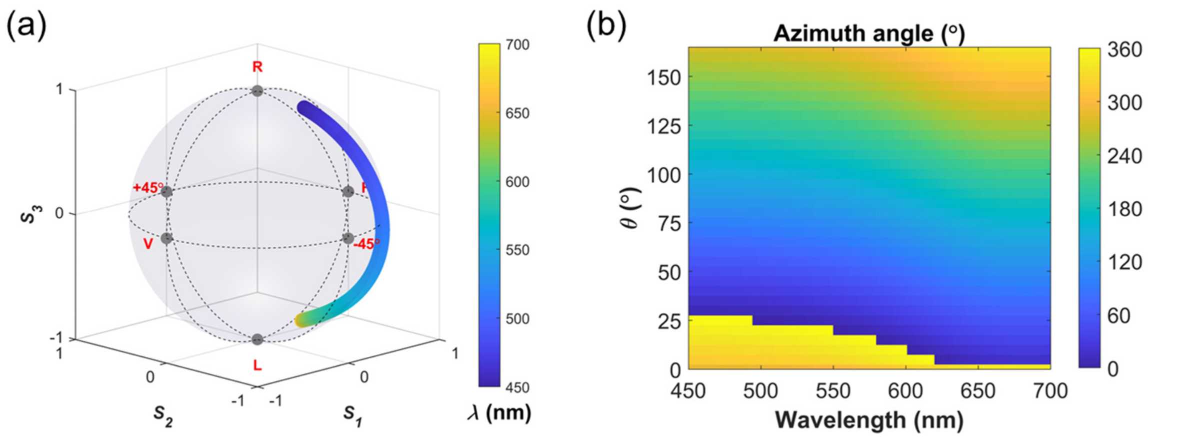

Figure S5. Simulated results showing (a) Stokes parameters on the Poincaré sphere as a function of wavelength and (b) the azimuthal angle as a function of both wavelength and structural rotation angle θ. In these simulations, the entire metasurface was rotated as a whole. The results closely match those presented in Figs. 2b and 3b in the main article, confirming that equivalent optical functionality can be achieved by rotating either individual meta-atoms or the entire metasurface.

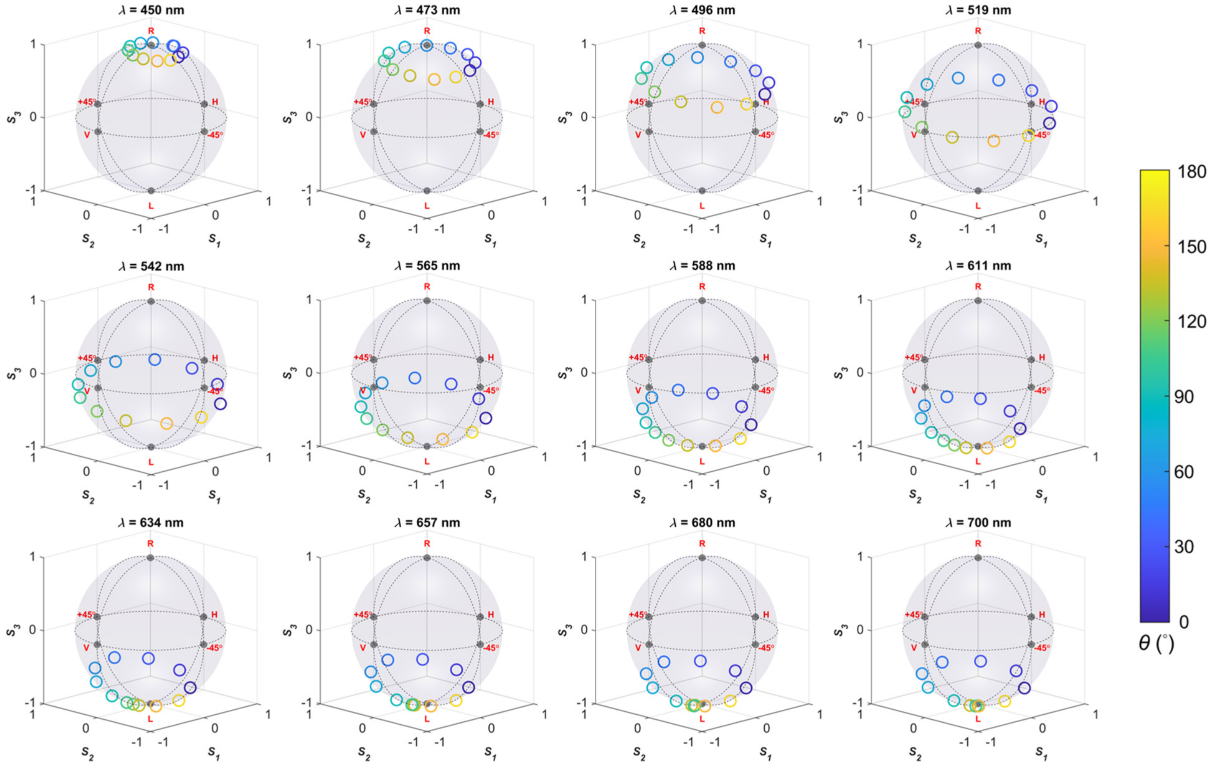

Figure S6. Stokes parameter distribution of the reflected light on the Poincaré sphere under varying wavelength conditions, highlighting the wavelength-independent nature of polarization control enabled by geometric polarization. These experimental results represent the average of measurements from three fabricated metasurface samples.

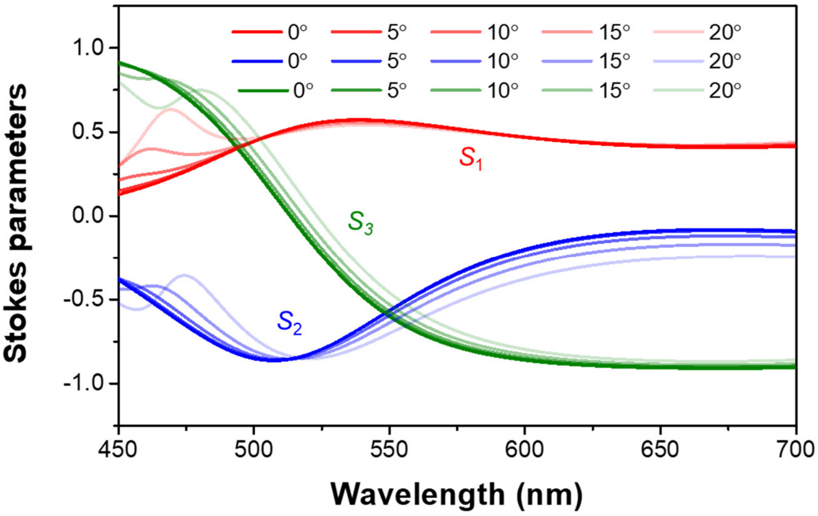

Figure S7. Simulated Stokes parameters as functions of wavelength and incident angle. The overall optical response remains largely unchanged when the angle of incidence is below 20°.

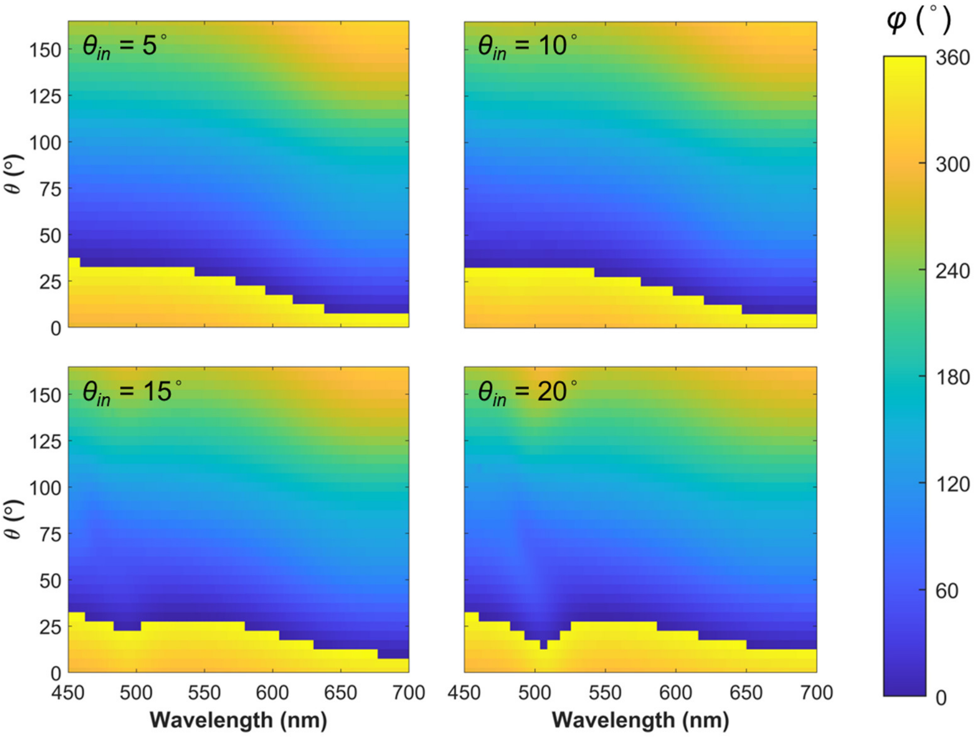

Figure S8. Simulated and measured azimuthal angles as functions of both wavelength and structural rotation angle θ under various angles of incidence θ_{in}. In the numerical simulations, individual meta-atoms were rotated about their central axes to evaluate the polarization response under different structural orientations. The geometric polarization response remains almost unchanged when the angle of incidence is below 20°.

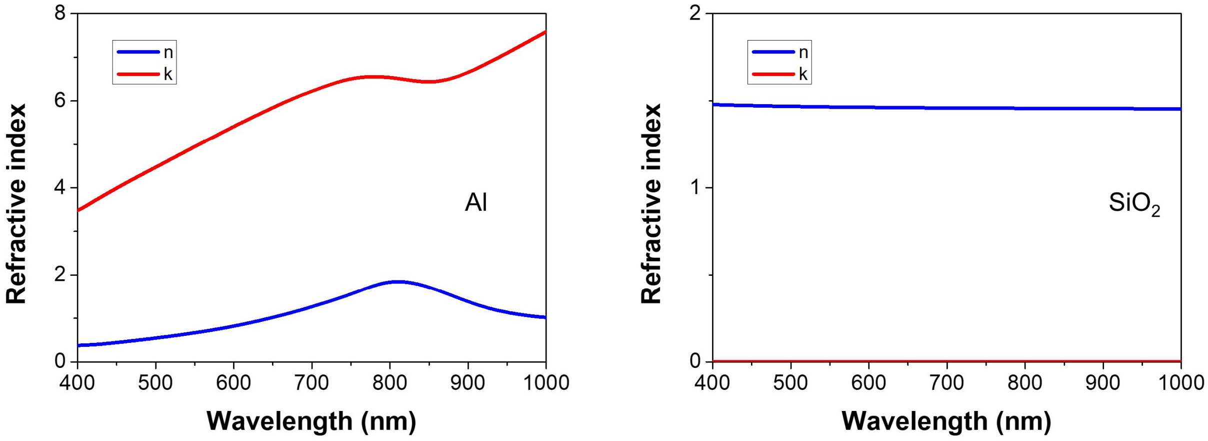

Figure S9. Experimentally measured refractive index of Al and SiO_2.

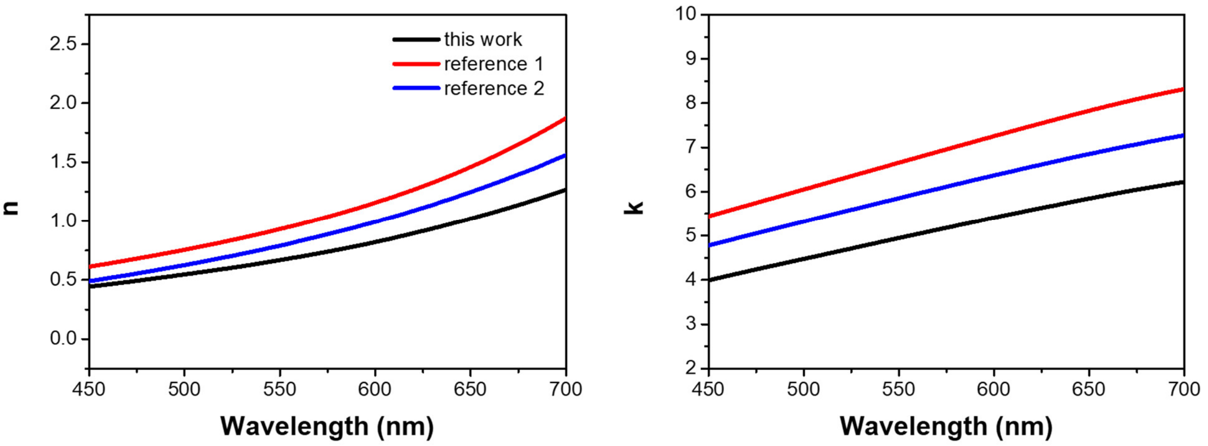

Figure S10. Experimentally measured refractive index of Al (black curves) and optical index obtained from Refs. 1 (red curves) and 2 (blue curves). The black curves were carried out from Fig. S9.

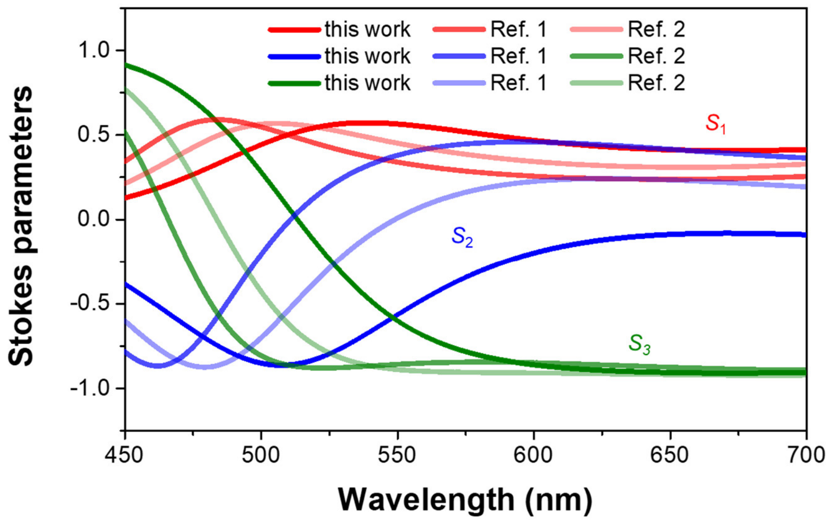

Figure S11. Numerically calculated Stokes parameters of the proposed metasurface using different optical indices of Al. The refractive indices labeled as "this work," "Ref. 1," and "Ref. 2" are extracted from the data presented in Fig. S10.

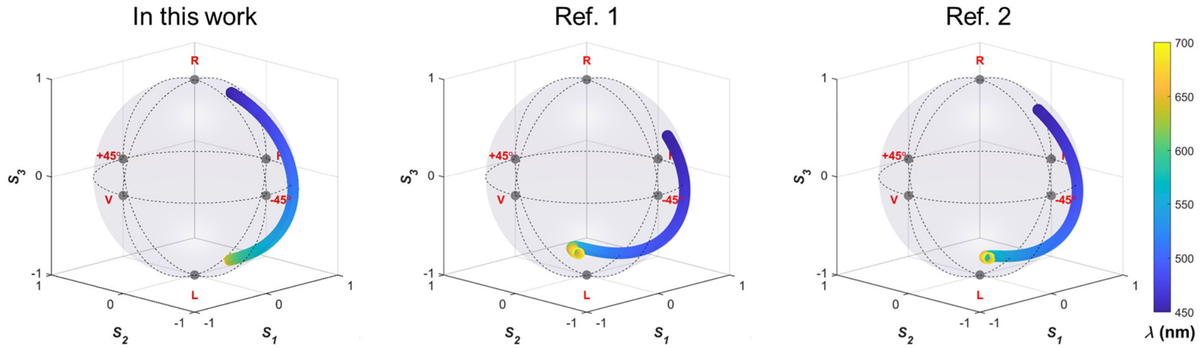

Figure S12. 3D plots of the simulated Stokes parameters on the Poincaré sphere using different optical indices of Al. The refractive indices labeled as "this work," "Ref. 1," and "Ref. 2" are extracted from the data presented in Fig. S10.

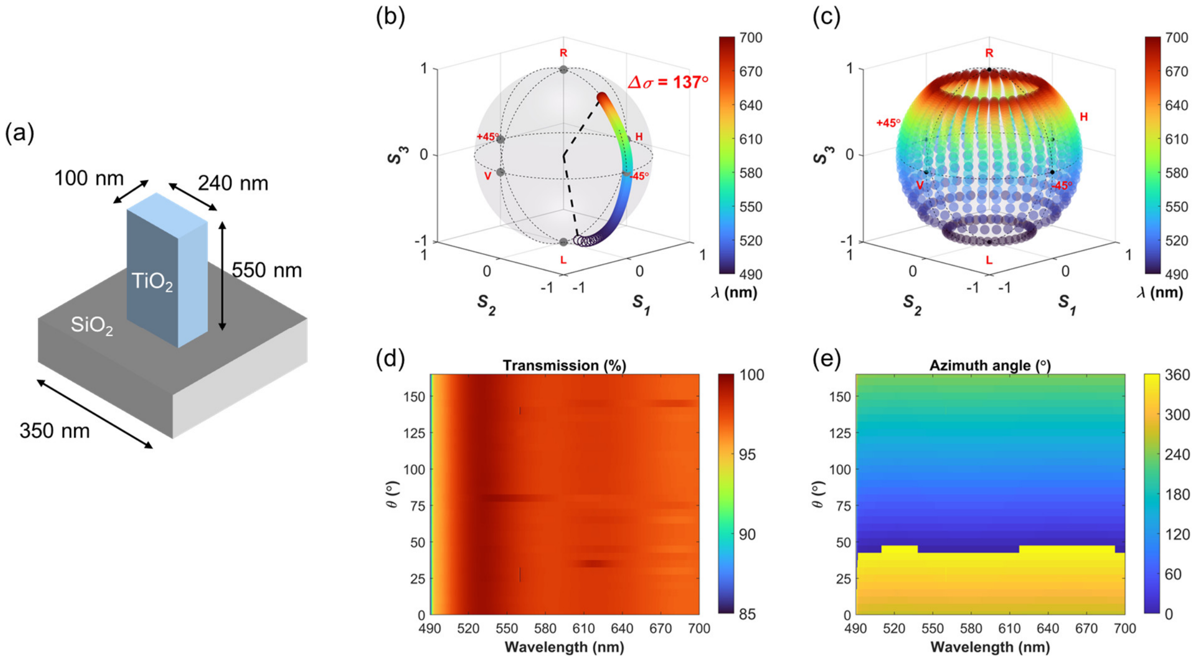

Figure S13. Demonstration of nearly full-Stokes polarization modulation using an all-dielectric metasurface in transmission. (a) Schematic illustration of the meta-atom composed of TiO2 nano-pillars on a glass substrate. (b) Simulated polarization trajectory on the Poincaré sphere as the wavelength varies from 490 nm to 700 nm, with the meta-atom orientation fixed. The result shows a continuous sweep across a wide latitude range with an azimuthal angle change of 137°, confirming broadband ellipticity modulation. (c) Combined spectral and rotational tuning produces a wide distribution of polarization states across the Poincaré sphere, indicating the feasibility of near full-Stokes polarization control in transmission. (d) Transmission map as a function of wavelength and rotation angle θ, showing consistently high transmittance (>90%) across the entire range. (e) Calculated azimuthal angle of the output polarization state, which varies smoothly with metasurface orientation and remains stable across the spectrum.

1. Derivation of Stokes parameter transformation under structural rotation with Jones matrix

To establish a rigorous theoretical foundation for our polarization modulation framework, we present here an explicit derivation that connects the output Stokes parameters to the co-polarized (r_{co}) and cross-polarized (r_{cross}) components of the optical response of the metasurface. We begin by considering a normally incident right-handed circularly polarized (RCP) electric field expressed, in the linear polarization basis as:

$$E_{in} = \frac{1}{\sqrt{2}}\begin{bmatrix} 1 \\ i \end{bmatrix} \tag{S1}$$

By applying the Jones matrix of the metasurface[3] to this input, the output field is obtained as:

$$\begin{bmatrix} E_x^{out} \\ E_y^{out} \end{bmatrix} = \left\{\frac{r_l+r_s}{2}\begin{bmatrix} 1 & 0 \\ 0 & 1 \end{bmatrix} + \frac{r_l-r_s}{2}\frac{1}{\sqrt{2}}\left[\begin{bmatrix} \cos 2\theta & sin2\theta \\ sin2\theta & -cos2\theta \end{bmatrix}\right]\begin{bmatrix} 1 \\ i \end{bmatrix}\right\} = \frac{r_l+r_s}{2\sqrt{2}}\begin{bmatrix} 1 \\ i \end{bmatrix} + \frac{r_l-r_s}{2\sqrt{2}}e^{i2\theta}\begin{bmatrix} 1 \\ -i \end{bmatrix} \tag{S2}$$

Here, r_l and r_s are the complex reflection coefficients of the meta-atom along its local fast and slow axes, respectively, and θ is the in-plane rotation angle of the meta-atom. After evaluating the matrix operation, the output field can be decomposed into circularly polarized basis states as:

$$\begin{bmatrix} E_x^{out} \\ E_y^{out} \end{bmatrix} = \frac{r_{\text{co}}}{\sqrt{2}}\begin{bmatrix} 1 \\ i \end{bmatrix} + \frac{r_{\text{cross}}}{\sqrt{2}}e^{i2\theta}\begin{bmatrix} 1 \\ -i \end{bmatrix} \tag{S3}$$

where r_{co} and r_{cross} represent the complex reflection amplitudes for the co- and cross-polarized circular components, respectively.

To derive the Stokes parameters, we first calculate the output field intensities and cross terms:

$$|E_x^{out}|^2 = E_x^{out} \times \text{conj}(E_x^{out}) = \frac{1}{2}\left\{|r_{\text{co}}|^2 + |r_{\text{cross}}|^2 + 2\text{Re}\left[r_{\text{co}} \cdot r_{\text{cross}}e^{-i2\theta}\right]\right\} \tag{S4}$$

$$\left|E_y^{out}\right|^2 = E_y^{out} \times \text{conj}\left(E_y^{out}\right) = \frac{1}{2}\left\{|r_{\text{co}}|^2 + |r_{\text{cross}}|^2 - 2\text{Re}\left[r_{\text{co}} \cdot r_{\text{cross}}e^{-i2\theta}\right]\right\} \tag{S5}$$

$$E_x^{out} \times \text{conj}\left(E_y^{out}\right) = \frac{-i}{2}\left(|r_{\text{co}}|^2 - |r_{\text{cross}}|^2\right) - \text{Im}\left[r_{co}\text{conj}(r_{\text{cross}})e^{-i2\theta}\right] \tag{S6}$$

where conj(q) represents the conjugation of the complex number q. The output Stokes parameters are then obtained using their standard definitions:

$$S_0 = |r_{\text{co}}|^2 + |r_{\text{cross}}|^2 \tag{S7}$$

$$S_1 = |E_x^{out}|^2 - \left|E_y^{out}\right|^2 = 2\text{Re}\left[r_{\text{co}} \cdot r_{\text{cross}}e^{-i2\theta}\right] \tag{S8}$$

$$S_2 = 2\mathrm{Re}\{E_x^{out} \times \mathrm{conj}(E_y^{out})\} = 2\mathrm{Im}[r_{\mathrm{co}}\mathrm{conj}(r_{\mathrm{cross}})e^{-i2\theta}] \quad \text{(S9)}$$

$$S_3 = -2\mathrm{Im}\{E_x^{out} \times \mathrm{conj}(E_y^{out})\} = (|r_{\mathrm{co}}|^2 - |r_{\mathrm{cross}}|^2) \quad \text{(S10)}$$

These expressions clearly reveal that the output polarization state, which is represented as a point on the Poincaré sphere, is determined by both the amplitudes and the relative phase between the co- and cross-polarized reflection components. The parameter S_3 corresponds to the intensity difference between these two components and describes the ellipticity or circular nature of the output polarization. In contrast, S_1 and S_2 arise from the coherent interference between the co- and cross-polarized components. These parameters are modulated by the complex reflection coefficients r_{co} and r_{cross} as well as the rotation angle θ, where the latter introduces a geometric phase through the cross-polarized channel. Since the geometric structure of the meta-atom remains unchanged throughout our study, both r_{co} and r_{cross} can be assumed to remain constant in magnitude as the metasurface is rotated. As a result, only the angle-dependent phase factor $e^{i2\theta}$ varies with θ, leading to modulation of S_1 and S_2 while leaving S_0 and S_3 unaffected. This behavior indicates that the polarization state traces a circle of constant latitude on the Poincaré sphere as the metasurface rotates. This theoretical prediction is in excellent agreement with our experimental results.

In addition to the angular modulation enabled by the geometric phase, another key feature of our approach lies in the wavelength-dependent response of the metasurface. Specifically, both the amplitudes of r_{co} and r_{cross} as well as the phase of r_{co} can be effectively tuned by varying the wavelength of the incident light. Through appropriate design of the meta-atom geometry and dimensions, the dispersion characteristics can be engineered such that different wavelengths within the operational bandwidth correspond to output polarization states that span a broad range along the longitudinal (i.e., meridional) axis of the Poincaré sphere. In practice, this means that by sweeping the input wavelength from the shortest to the longest value within the design range, the polarization state can be continuously transformed from near one pole of the Poincaré sphere to the opposite pole. This spectral modulation, when combined with in-plane structural rotation that introduces geometric phase control, provides two orthogonal and independent degrees of freedom. The result is an extended tuning capability that, in principle, allows the metasurface to achieve

nearly full coverage of the Poincaré sphere, and thus enables full-Stokes polarization modulation using a single-layer, non-interleaved metasurface platform.

2. **Transformation of Stokes parameters and azimuthal angle under structural rotation with the rotated coordinate frame**

When the anisotropic meta-atom is rotated by an angle θ, the electric field in the rotated frame (E'_x, E'_y) transforms as:

$$\begin{pmatrix} E'_x \\ E'_y \end{pmatrix} = \begin{pmatrix} \cos\theta & -\sin\theta \\ \sin\theta & \cos\theta \end{pmatrix} \begin{pmatrix} E_x \\ E_y \end{pmatrix} \quad \text{(S11)}$$

This corresponds to a passive rotation of the coordinate system (or, equivalently, an active rotation of the structure), which modifies the observed polarization state. If we substitute the rotated fields into the original Stokes definitions, it yields:

(i) Transformation of S_1

$$S'_1 = |E'_x|^2\text{-}|E'_y|^2 = (E_x\cos\theta - E_y\sin\theta)(E_x^*\cos\theta - E_y^*\sin\theta) - (E_x\sin\theta + E_y\cos\theta)(E_x^*\sin\theta + E_y^*\cos\theta) = \left(|E_x|^2\cos^2\theta + |E_y|^2\sin^2\theta - 2\sin\theta\cos\theta\,\mathrm{Re}(E_xE_y^*)\right) - \left(|E_x|^2\sin^2\theta + |E_y|^2\cos^2\theta + 2\sin\theta\cos\theta\,\mathrm{Re}(E_xE_y^*)\right) = \cos 2\theta\left(|E_x|^2 - |E_y|^2\right) - 2\sin 2\theta\,\mathrm{Re}(E_xE_y^*) = S_1\cos 2\theta - S_2\sin 2\theta \quad \text{(S12)}$$

(ii) Transformation of S_2

$$S'_2 = 2\mathrm{Re}(E'_xE'^*_y) = 2\mathrm{Re}\left((E_x\cos\theta - E_y\sin\theta)(E_x^*\sin\theta + E_y^*\cos\theta)\right) = 2\left(\sin\theta\cos\theta\,|E_x|^2 + \mathrm{Re}(E_xE_y^*)\cos^2\theta - \mathrm{Re}(E_xE_y^*)\sin^2\theta - \sin\theta\cos\theta\,|E_y|^2\right) = S_1\sin 2\theta + S_2 cos\, 2\theta \quad \text{(S13)}$$

(iii) Transformation of S_3

$$S'_3 = \text{-}2\mathrm{Im}(E'_xE'^*_y) = \text{-}2\mathrm{Im}\left((E_x\cos\theta - E_y\sin\theta)(E_x^*\sin\theta + E_y^*\cos\theta)\right) = 2\left(\mathrm{Im}(E_xE_y^*)\cos^2\theta + \mathrm{Im}(E_xE_y^*)\sin^2\theta\right) = S_3 \quad \text{(S14)}$$

Thus, the circular polarization component (associated with S_3) is invariant under in-plane structural rotation. Only the linear components S_1 and S_2 rotate in the Stokes space.

Using the transformed S'_1 and S'_2, the azimuthal angle φ of the polarization state (defined as the orientation of the linear polarization) undergoes the following transformation:

$$\varphi = \tan^{-1}\left(\frac{S_2}{S_1}\right) \rightarrow \varphi' = \tan^{-1}\left(\frac{S'_2}{S'_1}\right) \quad \text{(S15)}$$

Substituting the expressions of S_1' and S_2':

$$\varphi' = \tan^{-1}\left(\frac{S_1 \sin 2\theta + S_2 cos\, 2\theta}{S_1 \cos 2\theta - S_2 \sin 2\theta}\right) = \tan^{-1}\left(\frac{S_1 \sin 2\theta + S_1 \tan\varphi \cos 2\theta}{S_1 \cos 2\theta - S_1 \tan\varphi \sin 2\theta}\right) = \tan^{-1}\left(\frac{\tan 2\theta + \tan\varphi}{1 - \tan\varphi \tan 2\theta}\right) \quad \text{(S16)}$$

This results in a standard trigonometric identity:

$$\varphi' = \varphi + 2\theta \quad \text{(S17)}$$

This linear relationship confirms that the azimuthal angle of the polarization vector increases by 2θ for each structural rotation by θ, independent of output ellipticity.

3. Methods

Simulation

Numerical modeling was conducted using the finite element method (FEM) implemented in the frequency-domain solver of CST Studio Suite. A single meta-atom with lateral dimensions of 250 nm × 250 nm was simulated under unit cell boundary conditions to emulate the optical behavior of an infinite metasurface array. The material dispersion data for Al and SiO_2 were obtained from ellipsometric measurements (refer to Fig. S9). Variations in the deposition conditions of Al can lead to differences in surface roughness and grain structure, which in turn may alter the optical properties of the resulting film[2, 4]. Since the optical response of the plasmonic metasurface primarily arises from the resonant modes of the nanostructures and their near-field interaction with the reflective substrate, discrepancies in the optical constants of aluminum can cause deviations between simulated and experimental results (see Figs. S10–S12 for details). To ensure that the simulations accurately reflect the experimental conditions, the refractive index of Al used in all numerical simulations in this work was obtained through ellipsometric measurements of the fabricated films.

Fabrication

The fabrication process begins with the formation of a reflective base layer by depositing a 120 nm aluminum film onto a Si substrate using a thermal evaporation technique. A dielectric spacer layer of silicon dioxide SiO_2, tailored to the design thickness, is then added via magnetron sputtering. A layer of PMMA A4 resist is applied by spin coating in two stages: the substrate is rotated at 600 rpm for 10 seconds to spread the resist, followed by a high-speed spin at 6000 rpm for 60 seconds to ensure uniform coverage. The resist-coated sample is soft-baked at 180 °C for 3 minutes to evaporate residual solvent. To prevent charging during electron beam lithography, a thin conductive polymer layer (Espacer) is coated onto the surface prior to pattern exposure. Electron beam lithography is performed using an Elionix ELS-7500 system to define the nanostructures. After exposure, the Espacer layer is washed off with deionized water. The exposed resist is then developed in a mixed solution of methyl isobutyl ketone (MIBK) and isopropyl

alcohol (IPA) at a 1:3 ratio for 2 minutes, and the development is stopped by immersing the sample in pure IPA for 30 seconds. Following the development process, a 30 nm Al layer is deposited via thermal evaporation at a controlled rate of 1 Å/s. The final metasurface structures are realized through a lift-off process in acetone, which removes the residual resist and leaves behind the Al meta-atoms.

Reference

(1) Palik, E. D. *Handbook of Optical Constants of Solids*; Academic, **1985**.

(2) McPeak, K. M.; Jayanti, S. V.; Kress, S. J. P.; Meyer, S.; Iotti, S.; Rossinelli, A.; Norris, D. J. Plasmonic films can easily be better: Rules and recipes. *ACS Photonics* **2015**, *2*, 326-333.

(3) Chen, W. T.; Zhu, A. Y.; Capasso, F. Flat optics with dispersion-engineered metasurfaces. *Nat. Rev. Mate.* **2020**, *5*, 604–620.

(4) Knight, M. W.; King, N. S.; Liu, L.; Everitt, H. O.; Nordlander, P.; Halas, N. J. Aluminum for plasmonics. *ACS Nano* **2013**, *8*, 834-840.
